# Exploring Long-Term Exercise and Fitness Maintenance Using Group Versus Individual Training in a Diverse Group of Breast Cancer Survivors

**DOI:** 10.3390/healthcare14111556

**Published:** 2026-06-02

**Authors:** Lindsey Merifield, Matthew Toyama, Ashley Gooman, Cheri Teranishi-Hashimoto, Eunjung Lim, Paulette M. Yamada, Jami A Fukui

**Affiliations:** 1 Department of Translational and Clinical Research, University of Hawai‘i Cancer Center, Honolulu, HI 96813, USA; lemerif@gmail.com; 2 Clinical Trials Office, University of Hawai‘i Cancer Center, Honolulu, HI 96813, USA; mtoyama@cc.hawaii.edu (M.T.); algooman@hawaii.edu (A.G.); 3 Outpatient Therapy Program, Women’s Health and Cancer Rehabilitation Hospital of the Pacific, Honolulu, HI 96720, USA; cheri.teranishi@rehabhospital.org; 4 Quantitative Health Sciences, University of Hawai’i at Mānoa, Honolulu, HI 96813, USA; lime@hawaii.edu; 5 Department of Kinesiology and Rehabilitation Science, University of Hawai‘i at Mānoa, Honolulu, HI 96822, USA; pyamada@hawaii.edu

**Keywords:** breast cancer survivors, exercise, strength, endurance, range of motion

## Abstract

Background: While exercise is critical for breast cancer survivors, identifying prescriptions that promote long-term adherence is a challenge. This study evaluated whether survivors could maintain fitness gains during a 6-month self-managed phase following two distinct 12-week supervised programs. Specifically, we compared whether transitioning from one-on-one training to either individualized (Ind) or group-based (Gr) sessions influenced a participant’s ability to sustain improvements in strength, range of motion (ROM), and cardiorespiratory endurance (VO_2_peak). Methods: Thirty breast cancer patients from diverse backgrounds completed an initial 12-week supervised individual training program. They were then randomized into either Ind (*n* = 13) or Gr (*n* = 17) supervised sessions for a second 12-week phase. Fitness assessments were conducted at baseline, 3 months (post-initial training), and 1 year (after the 6-month self-managed phase). Data were analyzed using generalized estimating equations to evaluate the effects of time and training format. Results: Significant improvements were observed across all fitness categories over time. Muscular strength (bench press, plank, and squats) and VO_2_peak increased significantly by 3 months and were successfully maintained at the 1-year follow-up mark. Shoulder ROM also showed significant improvement at 1 year. Notably, the training format (group vs. individual) had no significant impact on these outcomes, with the exception of one ROM metric. Conclusions: Initial supervised exercise leads to significant fitness gains that breast cancer survivors can successfully maintain for at least six months through self-management. These gains are sustained regardless of whether the preceding supervised training was delivered in a group or individual format, suggesting flexibility in clinical exercise prescriptions.

## 1. Introduction

Breast cancer accounts for approximately 30% of cancer diagnoses in women and has a rapidly growing survivor population which has made survivor-specific care a clinical priority [1,2]. Survivors face elevated risks of cardiovascular disease, metabolic syndrome, and mobility impairments [3,4,5,6]. Specifically, exposure to anthracyclines, trastuzumab, and aromatase inhibitors is linked to cardiotoxicity and hypertension, while chemotherapy often exacerbates weight gain and adipose tissue accumulation [2,4,7]. Given these risks, integrating supplementary cardiac care and lifestyle interventions into survivorship plans is essential.

Exercise is a potent intervention for reducing overall morbidity and mortality among cancer survivors [8,9,10]. The American College of Sports Medicine (ACSM) recommends that breast cancer survivors should perform a minimum of 150 min of moderate-intensity exercise per week [2]. While 8-to-12-week supervised programs significantly improve muscular strength, shoulder function, and cardiovascular health, supervised interventions consistently outperform unsupervised or home-based alternatives [7,11,12,13,14], Despite these short-term gains, the long-term maintenance of these benefits—defined here as six months or longer—remains poorly understood. The current literature lacks definitive “gold standard” prescriptions for ensuring lifelong exercise adherence [15].

Previous studies have shown that long-term exercise adherence is positively associated with muscular strength and aerobic capacity [16], exercise knowledge and beliefs about planned behaviors [17], and exercise history [18], whereas lower baseline physical activity was negatively associated with exercise adherence [15]. Previous work suggests that it could take at least 6 months for overweight, non-exercisers to learn how to maintain a new physically active lifestyle [19]. This study utilizes Habit Theory and a stepped-care approach to address the challenge of long-term adherence. Habit Theory suggests that behavior modification requires consistent repetition; while new habits may form in approximately 66 days, complex lifestyle changes often require two to six months to become sustainable [20,21]. Furthermore, meta-analyses suggest that successful maintenance interventions typically involve supervised elements, action planning, and social support [22].

A critical gap in existing research is the lack of ethnic diversity, as most clinical trials have focused on White participants [15]. This excludes minority populations who experience higher rates of recurrence and metabolic disease [23,24,25]. By including a diverse participant pool, this study aims to enhance the generalizability of findings to mixed-ethnic populations and address known disparities in survivorship outcomes. In a recent study of breast cancer patients in Japan, looking at patient education vs. a 4-month exercise program, showed no significant differences in the recreational physical activity level at 1 year compared with usual care, although some significant improvements in body composition, including body weight, body mass index, and body fat percentage, were observed [26]. Despite evidence of short-term improvements, it is unclear if there is sustained long-term outcomes from prior studies. A majority of studies do not capture long-term follow up for breast cancer outcomes. The primary objective of this study was to determine if a diverse sample of breast cancer survivors could self-manage their exercise programs to maintain or improve fitness (muscular strength, range of motion, and VO_2_peak) for six months following two consecutive 12-week supervised phases. To investigate the optimal format for fostering autonomy, a sub-aim compared whether individualized (Ind) or group-based (Gr) training—provided after an initial one-on-one intensive phase—differentially affected a participant’s ability to maintain fitness during the subsequent six-month self-management period.

## 2. Methods

This study was carried out in collaboration with an ongoing study at a rehabilitation center in association with the university Kinesiology and Rehabilitation Sciences department. The collaborating study integrates exercise interventions in cancer patients and survivors of all types of cancer. However, our study is only in breast cancer survivors that were eligible based on the criteria below. Patients needed to sign a separate consent to participate in our study. This ensured that both protocols maintained independence.

### 2.1. Participants

**Figure 1:**
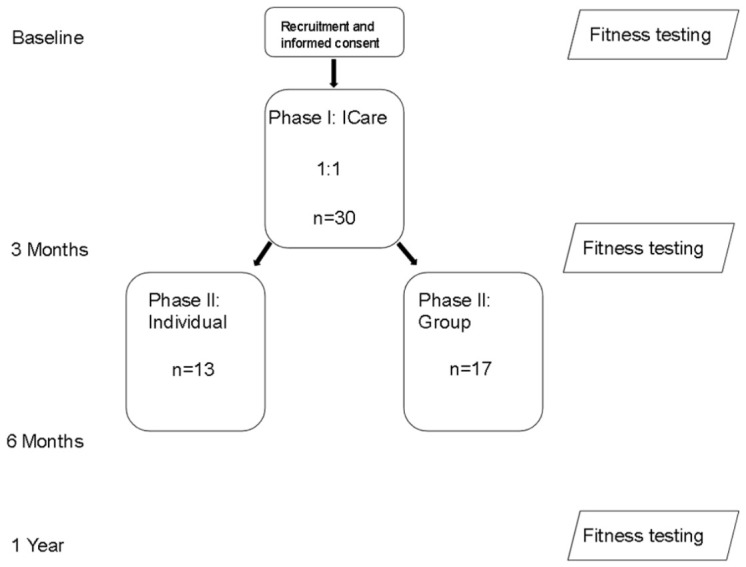
Study design flowchart.

Participants were recruited via oncologist referrals. The study was approved by Western IRB (IRB00000279) and registered under clinical trial number NCT04013568. Inclusion criteria included a diagnosis of any type of early-stage breast cancer within the past two years. Patients diagnosed with stage I, II, or III breast cancer who underwent surgery, chemotherapy, radiation, and/or endocrine therapy qualified for the study. Eligibility was determined by past medical history review and subjective medical history review conducted by a breast oncologist. Additionally, all participants underwent exercise clearance from their physician. Patients were not eligible if they had metastatic breast cancer (stage IV), pregnancy, uncontrolled psychiatric disorders that may affect self-assessment, recurrent breast cancer, and internal metal hardware (e.g., pacemaker, internal fixation, arthroplasty). Participants consented to participate after they were informed about the study visit schedule, exercise requirements, and required evaluations. Following informed consent and exercise clearance, all participants underwent baseline testing. Figure 1 depicts the study flow chart.

### 2.2. Intervention Design

The exercise intervention was divided into three stages. After baseline testing, participants completed an initial 12-week exercise program (90 min sessions, three times a week). Breast cancer survivors who maintain a healthier lifestyle are more likely to recognize the benefits of exercise, making them more likely to adhere to long-term fitness [15]. Thus, participants received an initial 12-week exercise program with a goal of helping participants to adopt a physical active lifestyle. Since perceived benefits of engaging in exercise could support intrinsic motivation [27], it was important for this 12-week intervention to provide individualized exercise training that would improve fitness while simultaneously providing education (e.g., exercise form, exercise principles, target intensity/exertion, learn how to differentiate between muscular pain associated with positive exercise adaptations and unexpected pain). Each session was led by a trainer with a background in cancer exercise rehabilitation. During this first stage of the study, all exercise sessions were delivered one-on-one with a trainer to ensure proper technique was used with individualized feedback.

In the second 12-week stage, participants were randomized using simple randomization to either grouped exercise sessions or continued with individual sessions. Randomization was allocated by a predetermined randomization chart provided by our statistician. The allocation was not concealed to provider, participant, or assessors given the nature of the sessions being individual or group. Both types of sessions were 90 min, twice a week. This design was employed because there is no universally accepted exercise prescription that promotes exercise adherence [15]; and the length of time or exercise format needed to support adherence is unknown. The current study design takes into account reported durations from 2 to 6 months for the development of lifestyle changes. In addition, this study design could provide additional information, e.g., if 6 months of exercise training is needed to support exercise adherence, could a more resource-efficient, group-based format be implemented?

The third stage (6 months to 1 year) had no exercise intervention program, but patients were advised to continue their fitness regimen. At the 1-year mark, strength, range of motion, and endurance were measured. Baseline, 3 month, and 1-year assessments were identical and included muscular strength (1 repetition max, 1RM), muscular endurance (chair squat test and plank), ROM (frontal and lateral raise), and cardiorespiratory endurance (VO_2_peak) measures.

### 2.3. Assessment Protocol

Muscular strength using seven different exercises was assessed with 1RM tests, including latissimus dorsi (lat) pulldown, seated cable row, incline bench press, shoulder press, leg extension, leg curl, and leg press. Patients were asked to lift as much weight as possible. If a 1RM was achieved, it was recorded. In cases where a 1RM was not attained, the amount of weight lifted and number of repetitions (up to 10) were used in the Brzycki Formula to estimate 1RM [28]. Lat pull down testing was performed with two cables on the barbell attachment. Seated cable row testing was performed with loose handle attachments. Inclined bench press and shoulder press testing was performed with an 18 lbs easy curl bar and weighted plates.

Lower body muscular endurance was measured with a chair squat test. Patients were asked to squat for as many repetitions as possible in 1 min. They were asked to place their arms across their chest and keep a rhythm as a pause in the exercise would be interpreted as resting and this would terminate the test. The depth of the squat was standardized with a chair and the number of repetitions was recorded (up to 60 s). Core muscular endurance was measured with plank holds. Patients were asked to hold a plank for as long as possible (no time limit).

Upper body flexibility was assessed with goniometry and range-of-motion (ROM) assessments. ROM was assessed in two different planes (lateral and frontal raise). Patients were asked to raise their arm, while keeping their straight elbow without shrugging. Each arm was assessed unilaterally.

VO_2_peak was estimated with the Shackelford Treadmill Protocol [28], which has been validated by the ACSM in 2009 as safe and effective for cancer patients. The last completed speed and grade were used with the ACSM equation to estimate VO_2_peak (walking VO_2_ = [(0.1 × S) + (1.8 × S × G) + 3.5], running VO_2_ = [(0.2 × S) + (0.9 × S × G) + 3.50, where speed (S) is in m/min and grade (G) is expressed as a decimal) [29]. The test was terminated once the patient reached volitional fatigue, or appearance of contraindications to exercise. During the test, patient heart rate, blood pressure, oxygen saturation, rate of perceived exertion, and heart rhythm (4-lead telemetric electrocardiogram) were monitored continuously. Patients were allowed to use handrails, and if they did, the VO_2_peak calculation was corrected for handrail use (adjusted VO_2_ = [(0.694 × estimated VO_2_) + 3.33]) [29].

A minimum of two fitness assessors conducted each fitness test. Assessors held Level 2 Clinical Cancer Exercise Specialist Certifications (University of Northern Colorado Cancer Research Institute), or were trained by certified personnel to conduct the fitness tests. Since assessors were involved in coordinating or providing exercise leadership, they were not blinded to study allocation. All tests were conducted with uniformity and feedback during testing was not provided to minimize bias.

### 2.4. Exercise Protocol

Exercise sessions were led by top-performing undergraduate Kinesiology and Rehabilitation Science students trained specifically in exercise oncology [30], under supervision by rehabilitation staff. All participants completed medical history questionnaires indicating previous injuries and medications that might affect exercise capabilities. Rehabilitation staff utilized this questionnaire in conjunction with baseline testing to create an individualized exercise prescription for each participant. Student trainers designed individualized training programs under faculty supervision, and instructed patients at each training session. Student training education practices were evaluated and found to be effective for both students and patients [30].

The exercise protocol followed Brown’s Protocol [31], which established four phases based on physical ability and medical history. Phase 1 was designed for participants undergoing chemotherapy/radiation treatment and prescribes the lowest intensity at 30–45% HRR (heart rate reserve) and 1RM, and RPE (rate of perceived exertion) 1–3. Phase 2 was designed for participants who had graduated from phase 1, completed chemoradiation, or had undergone only surgical/hormonal treatment. This phase used moderate intensity, at 40–60% of HRR and 40–60% of 1RM, and an RPE of 3–6. Phase 3 was for participants who had graduated from phase 2 (60–85% HRR, 60–85% of 1RM, RPE 4–8). Exercise sessions included 30 min of cardiovascular endurance, and 45 min of combined balance, resistance, and flexibility training. In accordance with the Principle of Progressive Overload, within the 12-week program, the intensity was increased each week and spanned the target range, e.g., a patient in phase 2 worked at 40% of HRR the first week, increased intensity throughout the program to reach 60% of HRR during week 12. Patients remained in the same phase for the 12-week one-on-on exercise training block. Patients who received one-on-one exercise sessions for both rounds progressed to the next phase for the second round. For example, a patient placed in phase 2 for the first round was advanced to phase 3 for the next round. Patients allocated to group-exercise training for the second round relied on RPE rather than prescriptive target heart rates. Exercise sessions were recorded to track patient progress.

Cardiovascular exercise included various modes of training like walking or jogging on a treadmill, upright/recumbent cycling, rowing, use of elliptical, and NuStep recumbent cross trainer. Modes were selected based upon patient preference and could be mixed and matched within a session for work bouts of a minimum duration of 10 min. Resistance training included open and closed chain exercises, and patients used equipment found in physical therapy clinics, e.g., machine weights, medicine balls, therabands, and Pilates equipment. Balance training was achieved through resistance training (e.g., split stance lunging) or trained separately (single leg balance). Flexibility was achieved through static or dynamic stretching, or specialized exercises like finger walks up a wall and use of postural equipment like the Fitness Cue.

All exercise sessions, both group and individual, were conducted at a local, licensed exercise rehabilitation facility. Grouped sessions utilized the same types of exercises as individualized sessions and had a ratio of 5 participants: 1 trainer. Patients were surrounded by other cancer survivors and supportive staff. As this study persisted during the COVID-19 pandemic, social distancing and online sessions were utilized to maintain social distancing requirements for a portion of the intervention.

### 2.5. Analysis

Demographic data were summarized as frequencies and percentages for the participants. Demographic differences were assessed with Pearson’s Chi-squared test and Fisher’s exact test for categorical variables, while Welch’s two-sample t-test was used to analyze continuous variables. To accommodate the sample size and non-normal distribution, and to correct for demographic differences between two sessions as needed, generalized estimation equations (GEE) were conducted with intervention, time, and their interaction on each variable, adjusting for within-subject variation. Analyses were performed for three time intervals: baseline, 3 months, and one year. Tukey–Sidak’s post hoc test was applied to assess pairwise differences for significant time effect. Results are reported as *p* values and mean± SD unless otherwise specified. Cohen’s d was calculated to measure the magnitude of the effect size for differences between time points, stratified by exercise session. The effect size is considered small if its absolute value is less than 0.2 and large if it is greater than 0.8 [32]. Statistical significance was set at *p* < 0.05 and all analyses were performed using R version 4.2.2 (Vienna, Austria).

## 3. Results

A total of 30 patients of mixed ethnic backgrounds were enrolled and completed all exercise training and follow-up testing with 100% adherence. The observed 100% adherence rate refers to the participants who completed the full 24-week protocol. This was supported by a flexible scheduling policy that allowed participants to make up missed sessions within an additional 12-week grace period before randomization. After 3 months, 17 were randomized to group training and 13 to individual training. No significant demographic differences were found between exercise type groups, including age, grade, surgery, chemotherapy, radiation, and ethnicity. The mean (SD) age of diagnosis for participants overall was 58 (9.9). Mean (SD) age was 60 (8.5) for group session participants and 54 (11.2) for individual session participants. (Table 1). At the time of data analysis, all participants completed all exercise sessions (100% attendance and adherence). In the case where participants were unable to attend, schedules were adjusted so that they exercised on a different day of the week. All exercise sessions were in person, enabling documentation of adherence. Each one-on-one exercise session was detailed in a dedicated patient logbook. Each week, logbooks were checked to ensure appropriate exercise intensity and duration and all components of fitness were targeted by an investigator (PY). Group-based exercise attendance was documented by the exercise leader where each session included an aerobic portion, resistance training, and flexibility. Participants gauged intensity with RPE; the exercise trainer provided real-time feedback to ensure appropriate exercise intensity. Post-exercise assessments were performed only after all sessions were completed. There were no adverse events from exercise participation. Although participants were randomized into groups, they were not evenly matched in terms of physical limitations. Post hoc chart analyses revealed that more individual session participants reported high severity shoulder pain, numbness, and stiffness.

### 3.1. Muscular Function

The exercise intervention resulted in improvements across nearly all strength metrics by the 3 months, including upper body, core, and lower body measures (*p* > 0.05, Table 2). However, there were no significant differences in outcomes between individual and group training formats, and notably, these fitness gains were successfully maintained at the 1-year follow-up point. While specific exercises like the leg press and leg curl showed more marginal results, the overall data indicate that both delivery methods are equally effective for fostering sustainable strength in breast cancer survivors.

Table 2 also shows the effect size for differences between time points, stratified by exercise session. The strength gains in the group sessions showed substantial long-term impact, characterized by large effect sizes (d > 0.88) for nearly all upper body, core, and primary lower body exercises from baseline to 1 year. Notably, the impact on the inclined bench press and shoulder press increased from medium at three months to large by the 1-year point. While the leg press and leg curl consistently maintained medium effect sizes throughout the study, the overall data highlight the robust and sustained clinical effectiveness of the group training format.

The strength improvements for participants in individual sessions were marked by significant clinical impact, with large effect sizes (d ≥ 0.84) sustained from baseline to the 1-year mark for the seated cable row, shoulder press, plank, and leg extension. While many other measures—including the lat pulldown, bench press, and leg press—maintained medium effect sizes over the full year, squats until fatigue showed a decrease to a small effect size. Overall, the data illustrate that individual training effectively drives powerful initial gains that remain clinically meaningful through long-term maintenance.

### 3.2. Range of Motion

No significant interaction was found between time and exercise type for any measures. A significance for exercise type was only found in the left lateral raise, where individual exercise sessions were higher than group sessions. Significant time differences were observed in the right frontal raise and left lateral raise, but no significant changes were found from baseline to 3 months or from 3 months to 1 year. At 1 year, the right frontal raise and left lateral raise were significantly improved from baseline (Table 2).

Regarding the effect size stratified by exercise session, medium effect sizes were found in group sessions from baseline to 3 months in the left frontal raise (Cohen’s d = 0.59), right frontal raise (Cohen’s d = 0.68), left lateral raise (Cohen’s d = 0.73), and right lateral raise (Cohen’s d = 0.5). From baseline to 1 year, large effect size was found in the right frontal raise (Cohen’s d = 0.94) and left lateral raise (Cohen’s d = 1.00). Medium effect size was found between baseline and 1-year left frontal raise (Cohen’s d = 0.66) and right lateral raise (Cohen’s d = 0.72) in group sessions (Table 2).

In individual sessions, a small effect size was found in the left frontal raise from baseline to 3 months (Cohen’s d = 0.17). Insignificant effect size was found in the right frontal raise (Cohen’s d = 0.01), left lateral raise (Cohen’s d = 0.10), and right lateral raise (Cohen’s d = 0.15) from baseline to 3 months. At 1 year, small effect sizes were found in the left frontal raise (Cohen’s d = 0.20), right frontal raise (Cohen’s d = 0.49), left lateral raise (Cohen’s d = 0.32), and right lateral raise (Cohen’s d = 0.24) in individual sessions (Table 2).

### 3.3. Endurance

No significant effects were observed by exercise type or their interaction, but a significant time effect was observed. No significant difference was found between 3 months and 1 year, but VO_2_peak significantly improved from baseline to 3 months, and was maintained at 1 year. A large effect size was found in both group (Cohen’s d = 1.30) and individual (Cohen’s d = 0.90) sessions from baseline to 3 months. From baseline to 1 year, the effect size remained large in both group (Cohen’s d = 1.43) and individual sessions (Cohen’s d = 1.26) (Table 2). The results for cardiorespiratory endurance (VO_2_peak) indicate that the exercise intervention provided a powerful and sustainable cardiovascular benefit for breast cancer survivors, regardless of the training format.

## 4. Discussion

In this pilot study, we explored if patients would be able to maintain their fitness over a 6-month period without exercise supervision. We found statistically and clinically relevant improvements in strength across upper body, core, and lower body measures from baseline to 3 months, with these gains being maintained at 1 year in both intervention groups. These findings align with a recent meta-analysis that reported that exercise interventions tended to have significant and moderate effects on the continuation of moderate-to-vigorous physical activity 6 months after an exercise intervention in breast cancer patients [33]; and that both group and individual physical activity interventions have positive adherence outcomes, with some indication that group interventions could have greater benefit in terms of psychosocial outcomes [34]. Self-reported reasons for exercise discontinuation included lack of time, exercise-related costs, and travel distance to the exercise facility [35], whereas, reasons for exercise continuation included the perception that training improved physical (less pain, better mobility) and psychological function (reduced stress, feeling good) [35].

Taking into account the findings from previous studies, results from the current study could suggest that the perceived benefits (physical and mental well-being) outweighed the time commitment. Also, since participants of the current study were asked to self-manage their exercise program, they selected the mode, intensity, frequency, and duration; and ultimately decided how to allocate their resources (cost, time, distance), which may have supported adherence. In the current study, exercise frequency decreased from 3×/week to 2×/week between the two rounds of exercise. Although a reduction in exercise frequency may have affected fitness gains, research has showed that resistance and aerobic training two or three times a week was associated with similar muscular adaptations [36] and aerobic capacity [37], respectively. On the other hand, frequency reduction may have promoted patients’ commitment to exercise as previous research has shown that time and travel distance could act as barriers to exercise [35].

Engaging in physical activity culturally rooted in a group’s culture could improve adherence to physical activity [38]. Hula, a culturally relevant form of physical activity, has been shown to improve fitness in a cohort of patients that share similar (ethnic/cultural) backgrounds to patients from the current study [39]. Participants of the hula study continued to hula ~6 months beyond the intervention. Their continued participation was attributed to their desire to stay connected and the strength of the relationships that they developed with one another [40]. While participants of the current study did not use a specific form of culturally relevant physical activity, their consistency could have helped to develop strong connections with training staff and their peers, and this form of community may have had a big role in determining adherence to the intervention and commitment to their fitness after the intervention.

Additionally, all strength measures that improved at 3 months maintained clinically relevant improvements at 1 year. The results of this study show that this intervention was associated with strength retention in breast cancer survivors for 6-months’ post-supervised training. This has substantial health implications for patients, representing that a 6-month training intervention results in long-term fitness effects and potential for decreased morbidities [41]. In combination with the evidence for positive health effects associated with improved fitness in breast cancer survivors, exercise training programs present a beneficial and scalable survivor care intervention.

Breast cancer survivors commonly experience discomfort, swelling, and/or weakness in the upper body following treatment [42]. Improvements in upper body strength and ROM may be especially helpful for quality of life, mobility, and activities of daily living (ADL), such as personal hygiene, household tasks, and other activities associated with independent living [43]. Decreases in strength are common after treatment, especially surgical interventions, and have been shown to improve with resistance training in this and previous studies [44,45,46]. Multiple other studies have reported increases in strength in aging [47] and cancer patients [48], suggesting improvements in strength are possible and beneficial across various populations and levels of fitness. Remodeling of skeletal muscle has also been shown to have benefits in chronic disease prevention through multiple metabolic responses and molecular mechanisms [41], reiterating the value of short-term preventative exercise training to breast cancer survivor health.

Furthermore, it is interesting to note that shoulder ROM significantly improved at 1 year, but not at the 3-month time point, suggesting that the continued self-managed exercise program facilitated ADLs involving overhead reaching. Improvements in shoulder mobility in this study are consistent with those of previous studies, which also associated increased shoulder range of motion with improved quality of life [49,50]. It is reasonable to infer that increasing range of motion may improve quality of life, as a previous study on functional shoulder movement found that 120 degrees of forward shoulder flexion and 130 degrees of shoulder abduction are required for ADL [48]. In this study, we demonstrated that shoulder mobility may require a longer time frame than strength for significant improvements. Notably, the majority of patients in this study exhibited a relatively high range of motion at baseline, which may partially explain the minimal short-term improvements. Previous exercise interventions in breast cancer patients found increased shoulder range of motion in the short term [48], but did not assess long-term changes. Increasing upper body mobility is especially important for breast cancer survivors as surgical intervention may cause discomfort, pain, edema, and decreased range of motion in survivors [51,52,53].

Increases in VO_2_peak from baseline to 3 months also showed clinical relevance and statistical significance. At 3 months, mean VO_2_peak increased to the “fair” range for women in their 50s per the ACSM guidelines [29]. Importantly, this improvement was maintained at 1 year. Increases in VO_2_peak represent increased aerobic capabilities mitigated by additional capillary formation, increased erythrocytes, and blood volume [54,55]. Decreases in oxygen consumption are a common issue for breast cancer survivors [56] and have been linked to poor health outcomes and increased mortality [29,57]. Increasing and maintaining oxygen consumption capabilities, as demonstrated in this study, has been associated with both improved quality of life and reduced mortality [57].

Previous studies have found similar results in VO_2_peak improvements [45,46,58,59,60]. Notably, all of these interventions were short term (8–16 weeks). Results at the 3-month mark align with the results found in short term studies, where statistically significant improvement in respiratory and endurance markers were measured. Since VO_2_peak is determined by oxidative capacity (arterial-venous oxygen difference) and cardiac output, current findings suggest that aerobic training improved oxygen utilization (mitochondrial function) and cardiovascular function, respectively. It is noteworthy to point out that the initial exercise stimulus was sufficient to induce improvements but more importantly, patients were able to maintain these gains on their own. Thus, initial exercise guidance and training do carry over a year-term, suggesting that providing a one-in-one 12-week intervention to educate patients about exercise programming followed by 12 weeks of either individualized or grouped-based training could be a scalable approach to help patients reduce overall mortality and morbidity risk over the long term.

Having the fitness to maintain independence in the workforce, family, and community contributes to broad, holistic impacts. This has the potential to translate to improved health of the community as a whole, e.g., survivors have the ability to fulfill family and occupational responsibilities, the venerable ability to teach and mentor younger generations, and participate in social settings [61]. This broad view puts the significance of long-term health into perspective, where not only is patient health important but equally so is helping individuals to maintain their capacity to contribute to society and lead fulfilling lives connected to their community and loved ones.

Cancer-specific fitness programs have reported high patient satisfaction and enthusiasm [62]. Integration of moderate-term fitness rehabilitation has the potential for multiple benefits, including drastic impact on cancer survivor health, hands-on experience for early career kinesiologists and researchers, and building connections within the cancer community. Unfortunately, cancer-specific fitness programs are not a standard component of survivor care. Establishing fitness programs as a regular practice in oncology, as outlined in the Team Kinesiology Model [63], may have considerable benefits to functionality-related quality of life, chronic disease prevention, psychological heath, and maintaining follow up in a scalable program [41,48,61,62,64].

### Limitations

A limitation of the study was that although participants were randomized into groups, they were not evenly matched in terms of physical limitations. Post hoc chart analyses revealed that more individual session participants reported high-severity shoulder pain, numbness, and stiffness. This could explain the smaller effect size in range of motion in individual session participants. Still, it is notable that those with shoulder mobility and pain issues still saw improvement in ROM, though less than those without prior shoulder complications. In other words, patients with fewer limitations would potentially have greater improvements. Future studies may consider variations in exercise limitations prior to randomization.

Due to funding limitations and collaboration with the existing program, fitness assessments were completed after the first round of exercise but not after the second round. From a research design standpoint, it was reasoned that even if a fitness test was administered after the second round of exercise, and fitness levels indeed increased, and then declined during the 6 months of self-exercise management, having these 6-month data would not be as critical as knowing the fitness of participants after the first round of exercise. This was a risk that was taken based upon the available resources. Results show that fitness was maintained from the 3-month to 12-month time point, indicating that participants had significantly improved fitness after the first round of exercise, and this level of fitness was maintained at the 1-year mark.

It is plausible that participants’ fitness further improved during the second round of exercise, and the final assessment captured a slow decline in fitness. Alternatively, participants may have not had significant fitness improvements after the second round of exercise and maintained this level of fitness. Additional research is needed to fully elucidate these mechanisms. Still, results from the current study show that participants were able to maintain fitness levels for 6-months’ post-supervised exercise training in a patient sample of mixed ethnic backgrounds. This is remarkable considering a similar study conducted in a cohort of Japanese breast cancer patients was unsuccessful in improving improved physical activity levels after a one-year period [26].

Also, the current study did not track participants’ fitness routine between 6 months and 1 year. Thus, although the findings show that fitness was maintained, it is not possible to show how participants were able to maintain these gains. This sequential design introduces potential confounding, making it difficult to isolate the effect of group vs. individual training. Future research should determine if a single, 12-week exercise intervention would be sufficient to support long-term adherence, and if group-based formats could be used instead of one-on-one, prescriptive exercise training.

Generalized estimating equations (GEE) was selected to evaluate population-averaged effects; however, we recognize that in a pilot sample of this size (N = 30), estimates may be sensitive. To ensure robustness, results were cross-verified for consistency, and we have interpreted marginal effects with caution. Future larger-scale studies may benefit from Linear Mixed Models (LMM) to further account for individual-level variability.

This study is characterized by a limited sample size, which reduces overall statistical power and the generalizability of the findings to the broader breast cancer survivor population. As a pilot exploration, the results should be interpreted cautiously. Additionally, while the sequential design allowed for a transition from supervised to self-managed exercise, it introduces potential confounding variables that may complicate the isolation of specific effects between group and individual training formats. In an effort to better serve the needs of the local breast cancer community, no control group was utilized in this study. Instead, the pre-intervention measures stood as baseline comparisons. Also, since participants self-elected to participate in the study, it can be reasoned that success may have been related to their baseline motivation or interest in exercising. Additionally, this study persisted during the COVID-19 pandemic, which required social distancing and online sessions for a portion of the intervention. Differences in exercise programming and equipment limitations due to online format may have affected the intervention; however, the instructor and types of physical activities were the same to maintain consistency as much as possible.

## 5. Conclusions

Overall, cardiovascular endurance and muscular strength showed improvement with exercise intervention that remained improved from baseline at 1 year (6-months’ post intervention) in both groups. Notably, range of motion showed improvement at 1 year, but not at 3 months, indicating that long-term training is needed to see significant improvements. The findings of this study suggest exercise intervention in breast cancer survivors can lead to sustained fitness improvements that have been linked to improved health outcomes by previous studies. Fitness improvements were maintained at 1 year and are linked to various health benefits. Considering this, creative ways for incorporating exercise programs should be considered for standardized survivorship care. Widespread use of fitness training for breast cancer patients and survivors, like the Team Kinesiology Model, could have vast impacts on survivor physical, mental, and functional health.

## Figures and Tables

**Table 1:** Participant demographics by exercise type.

Variable	Overall, *n* = 30	Exercise Type		*p*
		Group, *n* = 17	Individual, *n* = 13	
Age at diagnosis	58 (9.9)	60 (8.5)	54 (11.2)	0.144
Tumor grade				0.156
1	9 (30.0%)	5 (29.4%)	4 (30.8%)	
2	12 (40.0%)	9 (52.9%)	3 (23.1%)	
3	9 (30.0%)	3 (17.6%)	6 (46.1%)	
Surgery				0.666
Lumpectomy	23 (76.7%)	14 (82.4%)	9 (69.2%)	
Mastectomy	7 (23.3%)	3 (17.6%)	4 (30.8%)	
Chemotherapy	15 (50.0%)	7 (41.2%)	8 (61.5%)	0.461
Radiation	24 (80.0%)	13 (64.7%)	11 (84.6%)	0.927
Endocrine therapy	27 (90.0%)	15 (88.2%)	12 (92.3%)	1.000
Ethnicity				0.110
Asian (not specified) *	12 (40.0%)	4 (23.5%)	8 (61.5%)	
Japanese	14 (46.7%)	9 (52.9%)	5 (38.5%)	
White	3 (10.0%)	3 (17.6%)	0 (0.0%)	
Unknown	1 (3.3%)	1 (5.9%)	0 (0.0%)	

Two sample *t* test was utilized for age analysis, chi squared test utilized for chemotherapy, radiation, and endocrine analysis. Fisher’s exact test was utilized for grade, surgery and ethnicity. * Asian (not specified) includes those who are part-Asian and/or otherwise not specified A.

**Table 2:** Endurance, strength, and range of motion measures by exercise session type at baseline, 3 months, and 1 year.

Variable	Group, *n* = 17					Individual, *n* = 13					*p*		
	Baseline	3 Month	1 Year	Cohen’s d12	Cohen’s d13	Baseline	3 Month	1 Year	Cohen’s d12	Cohen’s d13	Time 12	Time 13	Time 23
***Strength***													
Lat pulldown (kg)	65.4 ± 8.3	74.8 ± 9.1	75.3 ± 8.8	1.52	1.52	67.6 ± 9.0	72.1 ± 13.2	72.4 ± 12.6	0.54	0.65	0.022	0.010	0.998
Seated cable row (kg)	58.5 ± 8.6	69.2 ± 9.0	72.9 ± 15.3	1.70	0.88	60.4 ± 9.3	67.6 ± 9.2	67.3 ± 10.4	1.31	1.10	<0.001	<0.001	0.915
Incline bench press (kg)	46.2 ± 12.2	57.6 ± 15.2	58.0 ± 13.0	1.49	1.54	45.1 ± 8.9	51.8 ± 16.7	51.8 ± 18.0	0.60	0.53	0.023	0.019	1.000
Shoulder press (kg)	36.3 ± 8.7	44.5 ± 10.4	46.3 ± 9.0	1.31	0.99	33.7 ± 6.7	42.3 ± 7.7	42.3 ± 9.8	1.06	0.84	<0.001	<0.001	0.977
Leg press (kg)	183.5 ± 49.3	215.7 ± 52.6	206.8 ± 40.0	0.75	0.60	187.7 ± 38.0	212.9 ± 59.0	203.1 ± 56.3	0.76	0.59	0.067	0.269	0.863
Leg extension (kg)	80.2 ± 26.4	107.3 ± 28.2	107.9 ± 28.0	0.98	0.87	77.2 ± 23.4	94.3 ± 32.4	97.5 ± 31.8	1.25	0.98	0.005	0.002	0.992
Leg curl (kg)	79.4 ± 18.2	90.8 ± 18.4	89.3 ± 16.5	0.73	0.62	74.3 ± 14.2	82.8 ± 15.2	84.5 ± 19.5	1.40	0.77	0.046	0.058	1.000
Squats until fatigue	32.9 ± 11.3	44.1 ± 15.2	45.7 ± 13.6	0.75	1.00	35.9 ± 13.5	42.5 ± 6.5	39.9 ± 10.2	0.50	0.29	0.009	0.018	0.998
Plank (seconds)	61.9 ± 39.5	136.1 ± 57.6	142.9 ± 59.7	2.46	1.94	89.2 ± 45.3	141.6 ± 78.4	143.5 ± 89.3	1.15	0.93	<0.001	<0.001	0.993
***Range of Motion***													
Left frontal raise (degree)	158.4 ± 14.6	165.1 ± 10.6	166.4 ± 7.4	0.59	0.66	159.8 ± 7.9	158.2 ± 7.0	162.8 ± 9.7	0.17	0.20	0.069	0.094	0.459
Right frontal raise (degree)	159.5 ± 12.1	166.5 ± 7.8	169.2 ± 9.3	0.68	0.94	162.5 ± 8.8	162.5 ± 8.5	167.5 ± 6.7	0.01	0.49	0.366	0.005	0.171
Left lateral raise (degree)	164.6 ± 9.0	171.8 ± 7.6	171.9 ± 7.9	0.73	1.00	172.1 ± 6.9	173.1 ± 10.1	175.6 ± 8.8	0.10	0.32	0.153	0.024	0.908
Right lateral raise (degree)	163.1 ± 12.3	169.8 ± 6.9	172.1 ± 9.2	0.50	0.72	169.5 ± 16.2	172.0 ± 8.7	173.9 ± 9.1	0.15	0.24	0.322	0.085	0.699
***Endurance***													
VO_2_peak (ml/kg/min)	27.9 ± 5.4	32.0 ± 4.3	33.7 ± 5.8	1.30	1.43	29.2 ± 6.1	35.1 ± 7.2	34.2 ± 4.5	0.90	1.26	0.003	<0.001	0.989

*p* value and means ± standard deviation. Generalized estimating equation (GEE) models were conducted with intervention, time, and their interaction on each variable. Interaction was not significant in any measure. Exercise type was significant in left lateral raise only (*p* < 0.01). For significant time effect, Tukey-Sidak’s post-hoc test was conducted to investigate significant differences between time points. No significant difference was found between groups in baseline patient characteristics. No patient characteristic corrections were made in GEE. 12 = baseline to 3 months. 13 = baseline to 1 year. 23 = 3 months to 1 year.

## Data Availability

The original contributions presented in this study are included in the article. Further inquiries can be directed to the corresponding author.
